# Temperature-assisted stabilization of aqueous polychlorinated biphenyl stock solutions for sorption experiments

**DOI:** 10.1016/j.mex.2026.103997

**Published:** 2026-06-09

**Authors:** Renáta Števuľová, Saimohana Krishna Vadlamudi, Ladislav Štibrányi, Ján Híveš

**Affiliations:** Slovak University of Technology, Faculty of Chemical and Food Technology, Radlinského 9, 812 37 Bratislava, Slovakia

**Keywords:** Polychlorinated biphenyls, Stock solution stabilization, Hydrophobic organic contaminants, Analytical reproducibility, Method development

## Abstract

Aqueous stock solutions of hydrophobic organic contaminants such as polychlorinated biphenyls (PCBs) are commonly used in laboratory sorption studies, yet their preparation and short-term stability are rarely described in sufficient detail. Because PCB congeners have high hydrophobicity and strong affinity for surfaces, apparent concentrations in aqueous stock solutions can change during storage and handling. This protocol describes a practical procedure for preparing, stabilizing, extracting, and analytically verifying aqueous PCB stock solutions prior to batch sorption experiments. The approach combines closed-bottle thermal equilibration, controlled hydrodynamic mixing, minimized plastic contact, sequential cyclohexane extraction, and congener-specific GC-ECD calibration. Validation using independently prepared stocks shows that heating and mixing increase the summed concentration of eight indicator PCB congeners after storage, while recovery testing demonstrates that two sequential extractions recover 94.8–99.5% of the cumulative three-extraction PCB concentration. The protocol is intended for laboratories working with technical PCB mixtures where reliable initial PCB concentration is required before sorption or kinetic studies.

## Highlights

• Provides a reproducible procedure for preparing and restabilizing aqueous PCB stock solutions prior to sorption experiments.
• Uses congener-specific external calibration for eight indicator PCBs and reports apparent aqueous concentrations rather than raw peak areas only.
• Includes sequential extraction recovery testing and practical guidance to minimize stock-solution variability.

## Graphical abstract

**Figure:**
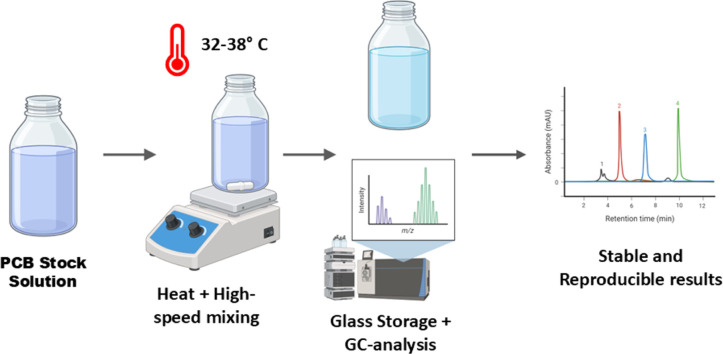

**Specifications table**

**Table:** 

**Subject area**	Chemistry
**More specific subject area**	Environmental Chemistry
**Name of your protocol**	Temperature-assisted stabilization of aqueous PCB stock solutions for sorption experiments
**Reagents/tools**	Technical PCB mixtures Delor 103 and Delor 106 (Chemko Strážske, Slovakia); PCB MIX-2 calibration standard in methanol (ANALYTIKA, spol. s.r.o., code CE151M, 1 µg mL^–1^ per congener); cyclohexane (HiPerSolv CHROMANORM, HPLC grade, ≥99.5% purity, VWR Chemicals/BDH Prolabo); distilled water; 2 L borosilicate glass bottles with borosilicate caps (Simax); 250 mL borosilicate glass vessels (Simax); VWR glass extraction vials with solvent-compatible snap-on lids; PTFE-compatible filtration where required; magnetic stirrer with PTFE-coated stir bars; IKA ETS-D5 temperature controller with probe; Thermo Scientific TRACE 1310 GC-ECD; TG-5SiMS column (30 m x 0.25 mm x 0.25 um)
**Experimental design**	Aqueous PCB stocks are stored in sealed borosilicate bottles, thermally equilibrated at 33 °C, mixed at 800–900 rpm, extracted with cyclohexane, and analyzed by GC-ECD using external calibration for eight indicator congeners. Two sequential cyclohexane extractions are recommended when quantitative recovery is required.
**Trial registration**	Not applicable
**Ethics**	This study did not involve human participants, animals or social media data.
**Value of the Protocol**	- Reduces hidden variability caused by storage and handling of aqueous PCB stocks. - Provides SOP-level extraction and GC-ECD quantification steps for eight indicator congeners. - Supports reproducible batch sorption experiments using technical PCB mixtures.

## Background

Aqueous stock solutions of polychlorinated biphenyls (PCBs) are routinely used in laboratory-scale sorption, photolysis, and remediation studies to prepare contaminated water matrices for kinetic and equilibrium experiments [[1], [2], [3]]. Their preparation is challenging because PCB congeners have high octanol–water partition coefficients, very low aqueous solubilities, and strong tendencies to partition to organic phases, container surfaces, colloids suspended microdomains, or other extractable fractions [[4], [5], [6]]. These properties can produce time-dependent changes in apparent aqueous concentration, particularly when stock solutions are stored before use or handled through multiple sampling and extraction steps.

Standard PCB analytical workflows commonly rely on solvent extraction of aqueous samples followed by gas chromatographic determination using electron-capture or other halogen-specific detection [[7], [8], [9]]. For example, EPA Method 3510C describes liquid–liquid extraction of water-insoluble and slightly water-soluble organic compounds from aqueous matrices, while EPA Method 8082A and Method 608.3 describe GC-based determination of PCBs in extracts [[7], [8], [9]]. These established methods provide analytical guidance for extraction and chromatographic determination, but they do not specifically address the pre-experimental stabilization of aqueous PCB stock solutions used in sorption or kinetic studies.

This distinction is important because sorption experiments often assume that the initial PCB stock concentration is stable and homogeneous before contact with the sorbent. In practice, however, stored PCB stocks prepared from technical mixtures may show concentration drift due to slow redistribution between the aqueous phase, bottle walls, headspace, stir bars, and any polymeric materials contacted during sampling. Such variability can affect calculated sorption capacities, kinetic trends, and removal efficiencies, especially when experiments are performed at low concentrations or repeated on different days.

**Fig. 1:**
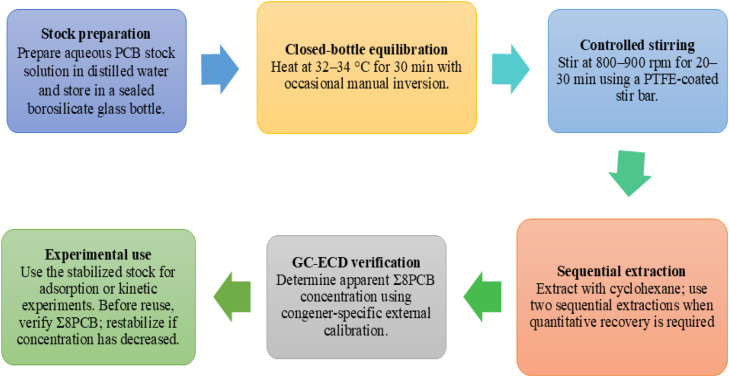
Workflow of the aqueous PCB stock solution stabilization protocol. The procedure combines sealed-bottle equilibration, controlled stirring, sequential cyclohexane extraction, and GC-ECD verification of apparent Σ8PCB concentration before use in sorption or kinetic experiments.

The present protocol addresses this practical gap by providing a standardized procedure for preparing, restabilizing, extracting, and analytically verifying aqueous PCB stock solutions prepared from technical Delor 103 and Delor 106 mixtures (Fig. 1). The workflow combines sealed-bottle thermal equilibration, controlled hydrodynamic mixing, minimized plastic contact, sequential cyclohexane extraction, and congener-specific GC-ECD calibration for eight indicator PCBs. The protocol is intended to improve reproducibility of PCB stock preparation before batch sorption experiments. It is not intended as a universal analytical method for all PCB formulations or hydrophobic organic contaminants; application to other mixtures, pure congeners, or concentration ranges should be separately validated.

## Description of protocol

### Rationale

**Fig. 2:**
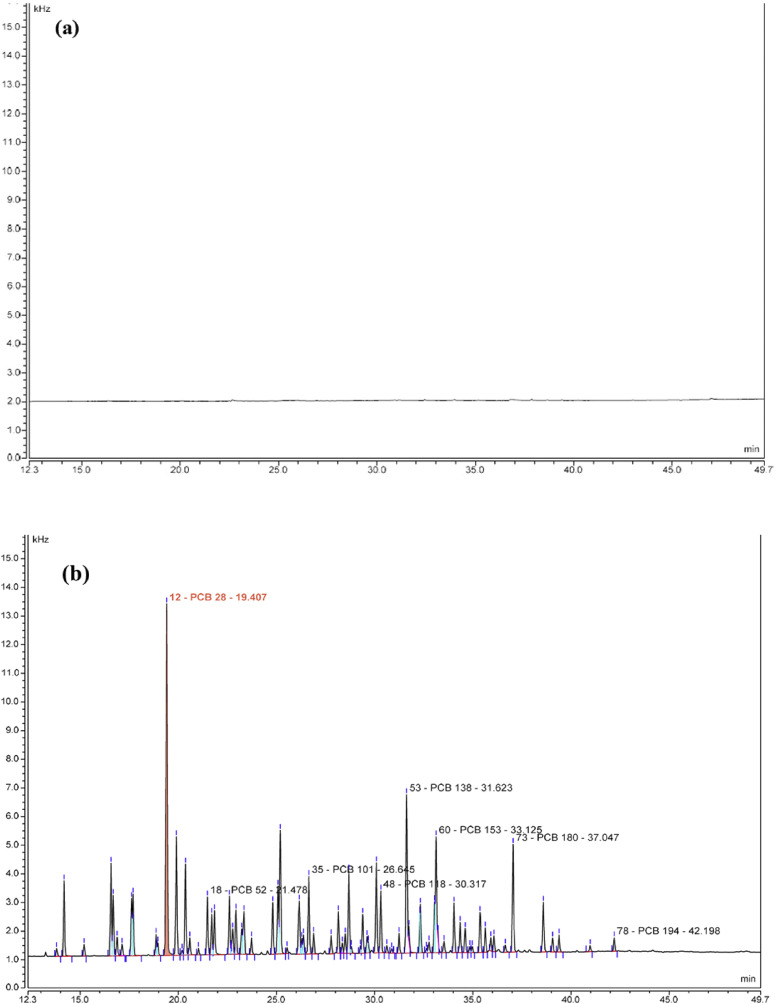

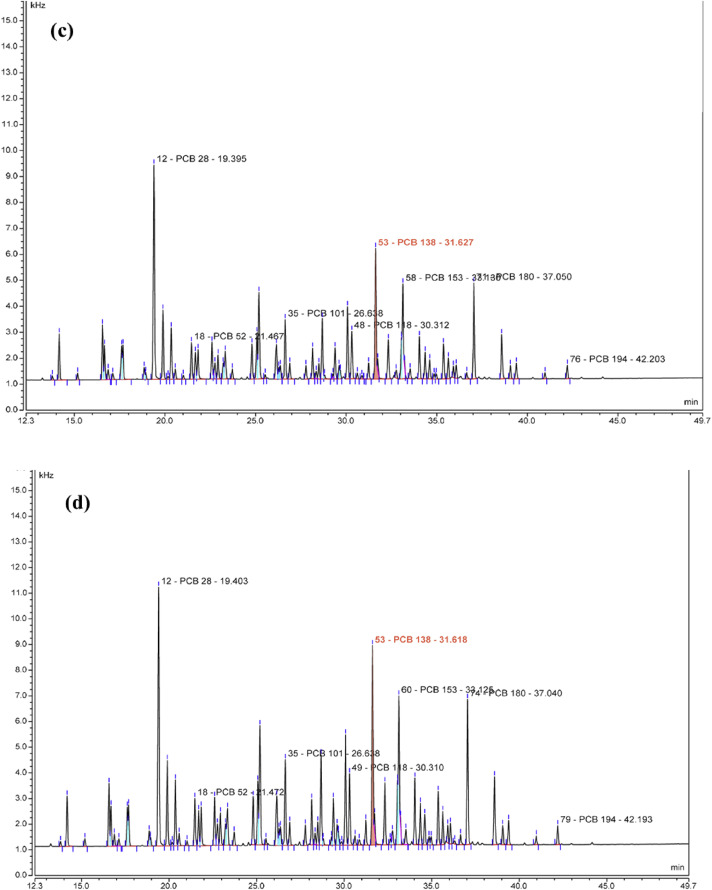
Representative GC-ECD chromatograms obtained during the preparation and verification of aqueous PCB stock solutions. (a) solvent/extraction blank; (b) original stock solution; (c) stock solution before stabilization; and (d) stock solution after stabilization using the proposed protocol involving controlled heating and mixing. The chromatograms show the characteristic retention-time positions of the monitored PCB congeners and illustrate the chromatographic profiles observed across the key stages of stock-solution preparation and stabilization.

Stored aqueous PCB stocks can show concentration drift because PCB congeners redistribute between the aqueous phase, glass surfaces, interfacial regions, and any polymeric materials contacted during sampling. This redistribution can introduce error into sorption experiments if the initial stock concentration is assumed rather than verified. The present protocol provides a practical workflow for restabilizing stored PCB stocks before use and for checking the resulting concentration of eight indicator congeners by calibrated GC-ECD analysis (Fig. 2).

The role of temperature in this protocol is primarily to accelerate stabilization of the prepared PCB-containing aqueous stock rather than to overcome the intrinsic low aqueous solubility of PCBs. PCBs are hydrophobic compounds with low water solubility, strong partitioning into non-polar phases, and substantial affinity for organic matter, particles, and container surfaces. Mild heating at 32–38 °C can increase molecular diffusion and interfacial mass transfer during mixing, thereby helping the PCB congeners redistribute more reproducibly between the introduced PCB mixture, the aqueous phase, and any operationally extractable microdomains present in the stock. Temperature-dependent solubility data for selected PCB congeners also show increased aqueous solubility between 5 and 35 °C, although the absolute solubilities remain low, especially for higher-chlorinated congeners. Therefore, the selected temperature range was used as a practical stabilization aid to improve reproducibility of the prepared stock solutions, while remaining low enough to avoid harsh thermal conditions, excessive volatilization, or degradation [10,11].

### Experimental design

The protocol includes six stages: (i) preparation and storage of aqueous PCB stock solutions in borosilicate glass, (ii) closed-bottle temperature-assisted equilibration, (iii) controlled magnetic stirring, (iv) sampling with minimized plastic contact, (v) sequential liquid-liquid extraction into cyclohexane, and (vi) GC-ECD analysis using external congener-specific calibration. The validation data were generated using three independently prepared stocks (A-C), and results are reported as apparent aqueous concentrations calculated from calibration curves.

### Reagents and tools

• Technical PCB mixtures Delor 103 and Delor 106 obtained from former production facilities of Chemko Strážske, Slovakia.
• PCB MIX-2 calibration standard in methanol (ANALYTIKA, spol. s.r.o.; code CE151M; 1 µg mL^–1^ of PCB 28, 52, 101, 118, 138, 153, 180, and 194).
• Cyclohexane (HiPerSolv CHROMANORM, HPLC grade, ≥99.5% purity; VWR Chemicals/BDH Prolabo) for liquid-liquid extraction.
• Distilled water as the aqueous matrix.
• 2 L borosilicate glass bottles with borosilicate glass caps (Simax, Czechoslovakia) for stock preparation and storage.
• 250 mL borosilicate glass reaction vessels (Simax) for sorption experiments.
• VWR glass extraction vials with solvent-compatible snap-on lids, with direct sample/extract contact with polymeric cap material avoided where possible.
• PTFE-coated magnetic stir bars and magnetic stirrer.
• IKA ETS-D5 temperature controller with probe for monitoring bath temperature.
• Thermo Scientific TRACE 1310 gas chromatograph equipped with electron-capture detector and TG-5SiMS fused-silica capillary column (30 m x 0.25 mm x 0.25 um).

Before use, glassware should be cleaned with laboratory detergent, rinsed thoroughly with distilled water, and rinsed with ethanol. Glassware should be dried before use. PCB handling, heating, extraction, and waste collection must be performed in a chemical fume hood with appropriate PPE.

Delor 103 and Delor 106 were used as the PCB sources for preparation of aqueous stock solutions. PCB MIX-2 was used as the external calibration standard for GC-ECD quantification of selected PCB congeners.

### Composition and physicochemical context of the PCB mixtures

Delor 103 and Delor 106 differ strongly in chlorination distribution. Delor 103 is dominated by lower-chlorinated homologues, whereas Delor 106 contains mainly higher-chlorinated homologues (Table 1). The indicator congeners monitored in this protocol cover different chlorination levels relevant to these mixtures (Table 2).

**Table 1:** Homologue composition of Delor PCB mixtures. Data compiled from published analyses of Delor technical mixtures [12,13].

Homologue Group	Delor 103 (%)	Delor 106 (%)
Di-chlorobiphenyls	10.4	0.02
Tri-chlorobiphenyls	53.8	0.5
Tetra-chlorobiphenyls	32.3	2.9
Penta-chlorobiphenyls	2.6	17.8
Hexa-chlorobiphenyls	0.3	52.9
Hepta-chlorobiphenyls	0.1	19.4
Octa-chlorobiphenyls	0	1.9
Nona-chlorobiphenyls	0	0.05

**Table 2:** Physicochemical properties of indicator PCB congeners. logKow and aqueous solubility values are compiled from published PCB physicochemical property datasets [4,5,11].

Congener	Number of Cl atoms	logKow	Aqueous solubility (mg l^–1^)
PCB 28	3	5.67	0.16
PCB 52	4	5.84	0.027
PCB 101	5	6.38	0.008
PCB 118	5	6.74	0.004
PCB 138	6	6.83	0.0009
PCB 153	6	6.92	0.0008
PCB 180	7	7.36	0.0004
PCB 194	8	7.80	0.00015

### Step-by-step protocol

**1. Preparation of aqueous PCB stock solution:** Add 1 mL of methanolic Delor 103 stock solution and 1 mL of methanolic Delor 106 stock solution to 1 L of distilled water in a 2 L borosilicate glass bottle with a borosilicate cap, leaving an approximate headspace of 1 L. Each methanolic Delor stock solution had a nominal PCB concentration of 1 mg mL⁻¹, giving a final nominal total PCB concentration of approximately 2 mg L⁻¹ and a final methanol fraction of approximately 0.2% v/v. Seal the bottle immediately after preparation and invert gently several times to disperse the methanolic spike. The same bottle volume, fill volume, closure type, and preparation procedure should be used for all stock solutions to keep headspace and handling conditions consistent.
Stock solutions A, B, and C were prepared independently from the Delor 103/Delor 106 PCB mixtures using the same temperature-assisted preparation procedure described in this protocol. These independently prepared stocks were used to evaluate the reproducibility of the stock preparation procedure. PCB MIX-2 was not used to prepare stock solutions A, B, and C; it was used only as an external analytical calibration standard for the selected indicator congeners quantified by GC-ECD (Table 3).

**Table 3:** External calibration equations for indicator PCB congeners.

Congener	Intercept	Slope	R^2^	Equation
PCB 28	0.00343	0.34509	0.996	Area = 0.00343 + 0.34509 x C_extract
PCB 52	0.00470	0.23364	0.986	Area = 0.00470 + 0.23364 x C_extract
PCB 101	0.00699	0.32684	0.986	Area = 0.00699 + 0.32684 x C_extract
PCB 118	0.01116	0.43923	0.979	Area = 0.01116 + 0.43923 x C_extract
PCB 138	0.01172	0.41108	0.980	Area = 0.01172 + 0.41108 x C_extract
PCB 153	0.01345	0.46210	0.978	Area = 0.01345 + 0.46210 x C_extract
PCB 180	0.01441	0.61644	0.982	Area = 0.01441 + 0.61644 x C_extract
PCB 194	0.01601	0.72006	0.978	Area = 0.01601 + 0.72006 x C_extract
**2. Storage before stabilization:** Store the sealed stock solution at ambient laboratory temperature (20–23 °C), protected from direct sunlight. Keep the bottle volume, fill volume, closure type, and headspace consistent between preparations, and open the bottle only briefly during sampling.
**3. Temperature-assisted equilibration:** Place the sealed bottle in a water bath maintained at 33 °C. Maintain this temperature for approximately 30 min. Gently invert the bottle every 5–10 min during heating. Temperature is monitored using the IKA ETS-D5 probe in the bath or a corresponding temperature-control setup; the stock bottle remains sealed during equilibration.
**4. Controlled hydrodynamic mixing:** After heating, stir the solution at 800–900 rpm for 20–30 min using a PTFE-coated magnetic stir bar while maintaining approximately 33 °C. Open-vessel heating or stirring under exhaust airflow is not recommended because it can promote volatile or interfacial losses.
**5. Sampling and sorption experiment setup:** For sorption experiments, transfer 100 mL of stabilized stock into 250 mL borosilicate glass vessels containing the sorbent material. Minimize plastic contact during sampling and filtration; use glass transfer devices where possible and PTFE-compatible components when filtration is required.
**6. Liquid-liquid extraction:** For stock verification, extract 50 mL of aqueous sample with 10 mL cyclohexane in a glass extraction vial at 1200 rpm for 10 min. Based on recovery testing, two sequential cyclohexane extractions are recommended when quantitative recovery is required. Thus, after collecting the first cyclohexane extract, extract the same aqueous phase again with fresh 10 mL cyclohexane under the same conditions. A third extraction is used only for recovery evaluation, not routine analysis.
**7. Restabilization before reuse:** If a stored stock is reused and the measured PCB concentration has decreased, repeat steps 3–6 before use. Standard restabilization is performed at 33 °C with stirring at 800–900 rpm for 20–30 min. Temperatures up to 38 °C may be used only as an upper experimental boundary in troubleshooting or stronger restabilization tests, and should be reported explicitly if used (Table 4).

**Table 4:** Experimental parameters used for temperature-assisted stabilization and restabilization of aqueous PCB stock solutions (heating temperature, mixing intensity, and equilibration time).

Step	Purpose	Temperature °C	Stirring in rpm	Time in min
Initial equilibration (heating only)	Promote short-term redistribution of PCBs prior to intensive mixing	32–34	Occasional inversion (Manual shaking)	30
Stabilization (heating + stirring)	Achieve short-term homogeneity and reproducible chromatographic response	32–34	800–900 rpm	20–30
Re-equilibration without heating (not recommended)	Test effect of mixing alone after storage	26 (room temperature)	800–900 rpm	10
Restabilization (moderate heating)	Restore chromatographic response after storage	32	800–900 rpm	15–30
Restabilization (stronger heating)	Further improve recovery when moderate heating is insufficient	38	Occasional inversion (Manual shaking)	15

### GC-ECD analysis and calibration

PCB congeners in cyclohexane extracts are analyzed using a Thermo Scientific TRACE 1310 GC-ECD fitted with a TG-5SiMS fused-silica capillary column (30 m x 0.25 mm x 0.25 um). Helium is used as carrier gas at a constant column flow of 1.0 mL min^–1^, corresponding to an inlet pressure of 85.56 kPa. Injection volume is 2 µL in split mode with a split ratio of 1:30. Injector and detector temperatures are 250 °C and 320 °C, respectively. The oven program is: 50 °C for 5 min, ramp to 320 °C over 50 min, and hold at 320 °C for 10 min, for a total run time of 66 min.

External calibration was performed using PCB MIX-2 standard (ANALYTIKA, code CE151M; 1 µg mL⁻¹ per congener in methanol). Calibration standards were prepared by serial dilution of PCB MIX-2 over dilution factors from 1:2 to 1:1000, corresponding to 0.001–0.5 µg mL⁻¹ per congener, and analyzed under the same GC-ECD conditions as the samples. Congener concentrations were calculated using linear equations of the form Area = intercept + slope × C_extract. Apparent aqueous concentrations were back-calculated from the extraction ratio. For 50 mL aqueous sample extracted into 10 mL cyclohexane, C_water = 0.2 × C_extract. When two sequential 10 mL cyclohexane extracts are analyzed separately from the same 50 mL aqueous sample, the total apparent aqueous concentration is calculated as C_water, total = 0.2 × (C_E1 + C_E2), where C_E1 and C_E2 are the congener concentrations measured in the first and second cyclohexane extracts, respectively.

### Data measurement and analysis

The primary response parameter is the summed apparent aqueous concentration of eight indicator PCB congeners (Ʃ8PCB): PCB 28, PCB 52, PCB 101, PCB 118, PCB 138, PCB 153, PCB 180, and PCB 194. In this protocol, apparent aqueous concentration refers to the extractable PCB amount per volume of sampled stock solution after standardized cyclohexane extraction; it should not be interpreted as the freely dissolved molecular concentration of each congener. Each congener is quantified from its calibration equation and then back-calculated to the apparent aqueous concentration using the extraction volume ratio. For sequential extraction recovery tests, recovery is calculated from the sum of congener concentrations in extract 1, extract 2, and extract 3. Values below the calibration intercept are treated as zero.

For the main validation dataset, three independently prepared PCB stock solutions (A-C) were analyzed. For the restabilization rows, Stock A was measured in triplicate; the Stock A mean was used together with single Stock B and Stock C measurements to calculate mean, standard deviation, and relative standard deviation across stocks.

### Analytical QA/QC

Quantification was performed using external congener-specific calibration with PCB MIX-2. Internal-standard correction was not applied because the available PCB MIX-2 standard contained the same congeners as the target analytes and was therefore suitable for external calibration but not for use as a non-target internal standard. Analytical reliability was supported by calibration linearity, solvent blanks, independent stock validation, and sequential extraction recovery testing.

Calibration curves were generated for all eight indicator PCB congeners with coefficients of determination ranging from 0.978 to 0.996. Blank cyclohexane injections should be run periodically to confirm the absence of carryover and background contamination. All extracts should be analyzed using the same GC-ECD method, integration settings, retention-time windows, and calibration tables. Sequential extraction recovery was evaluated using three consecutive cyclohexane extractions of representative aqueous stock samples. Plastic contact should be minimized throughout sampling and extraction because PCB partitioning to polymeric materials can introduce variability.

### Safety and regulatory considerations

All PCB handling, solution preparation, heating, stirring, extraction, and waste disposal must be performed inside a chemical fume hood using appropriate PPE. PCB-containing liquids, glassware rinses, used cyclohexane, and contaminated consumables must be collected as hazardous waste according to institutional and national regulations for persistent organic pollutants. This protocol does not involve human participants, animals, social media data, FDA-regulated testing, or clinical guidelines.

### Protocol validation

The protocol was validated using independently prepared aqueous PCB stock solutions subjected to storage, heating, heating plus mixing, and restabilization (Fig. 2). Concentrations were calculated from congener-specific external calibration curves and expressed as apparent aqueous Ʃ8PCB concentrations.

The concentrations reported in Table 5 should be interpreted as operationally determined extractable concentrations of the selected PCB congeners in the prepared aqueous PCB stocks, not as confirmation that all detected PCBs were present exclusively as freely dissolved molecules at thermodynamic equilibrium. This distinction is important because the summed concentration of the selected congeners can exceed literature aqueous solubility values for individual higher-chlorinated PCBs. PCBs are hydrophobic compounds with low aqueous solubility and can partition into organic phases, container surfaces, colloids, suspended microdomains, or other operationally extractable fractions. Therefore, the standardized preparation and solvent-extraction procedure provides a practical analytical measure of the PCB fraction present in the prepared aqueous stock, but it does not independently determine the freely dissolved concentration. The purpose of the protocol is to prepare reproducible PCB-containing aqueous stocks for subsequent sorption experiments. Studies requiring thermodynamically defined freely dissolved PCB concentrations should include an additional verification step, such as passive sampling or SPME.

**Table 5:** Operationally extractable concentrations of selected PCB congeners in prepared aqueous PCB stocks. Values represent solvent-extractable PCB concentrations measured after the described preparation and extraction procedure and should not be interpreted as independently verified freely dissolved equilibrium concentrations.

Condition	Stock A (mg l^–1^)	Stock B (mg l^–1^)	Stock C (mg l^–1^)	Mean (mg l^–1^)	SD	RSD (%)
Initial stock	1.36	1.34	1.36	1.35	0.010	0.8
After storage / before stabilization	1.05	0.880	0.964	0.966	0.087	9.0
Heating only	1.13	1.10	1.19	1.14	0.044	3.9
Heating + mixing	1.51	1.39	1.50	1.46	0.068	4.6
Before restabilization	0.873	0.823	0.884	0.860	0.032	3.8
After restabilization	1.49	1.30	1.45	1.41	0.100	7.1

The validation dataset in Table 5 was calculated from the first cyclohexane extraction performed consistently across all conditions. Sequential extraction was evaluated separately to determine extraction recovery and to define the recommended procedure for quantitative recovery.

Across the three independently prepared stocks, storage reduced the mean Ʃ8PCB concentration from 1.35 mg l^–1^ to 0.966 mg l^–1^. Heating alone increased the mean concentration to 1.14 mg l^–1^, while heating combined with stirring increased it to 1.46 mg l^–1^. Restabilization increased the mean concentration from 0.860 mg l^–1^ before restabilization to 1.41 mg l^–1^ after restabilization. These data support the use of closed-bottle heating and controlled stirring before sorption experiments.

Because occasional low PCB153 responses were observed in some individual chromatograms, a sensitivity check was performed by recalculating the summed concentration after excluding PCB153. The resulting Ʃ7PCB dataset showed the same qualitative trend as the Ʃ8PCB dataset, confirming that the observed stabilization effect was not controlled by a single congener.

Sequential extraction testing showed that a single cyclohexane extraction recovered 71.1–87.1% of the cumulative three-extraction Ʃ8PCB concentration, whereas two sequential extractions recovered 94.8–99.5%. Therefore, two consecutive cyclohexane extractions are recommended when quantitative recovery is required (Table 6). This recovery test was used to support the recommended two-step liquid–liquid extraction procedure for stock-solution verification and was not intended as a full regulatory-style recovery validation across multiple concentration levels, matrices, or surrogate/internal-standard conditions.

**Table 6:** Sequential extraction recovery calculated from congener-specific concentration conversion. E1, E2, and E3 represent the first, second, and third consecutive cyclohexane extractions of the same aqueous phase.

Condition	E1 Ʃ8PCB in extract (mg l^–1^)	E2 Ʃ8PCB in extract (mg l^–1^)	E3 Ʃ8PCB in extract (mg l^–1^)	Apparent aqueous total (mg l^–1^)	E1 recovery (%)	E1+E2 recovery (%)
Before restabilization - Stock A	5.26	0.750	0.027	1.21	87.1	99.5
After restabilization - Stock A	7.23	2.42	0.526	2.04	71.1	94.8

A supporting lower-concentration check at 0.5 mg l^–1^ also showed the same qualitative stabilization trend, with Ʃ8PCB chromatographic response increasing after stabilization. This supporting check should be interpreted as an additional concentration robustness observation rather than a full concentration-range validation.

Storage experiments also showed that PCB losses were higher in plastic containers than in borosilicate glass containers, confirming stronger sorption to polymer surfaces.

## Limitations

The protocol was developed and validated for aqueous stock solutions prepared from technical Delor 103 and Delor 106 PCB mixtures under the tested laboratory conditions. It improves short-term stock homogeneity and extraction reproducibility but does not increase the intrinsic aqueous solubility of PCB congeners (Table 7). Therefore, measured apparent concentrations may include freely dissolved, dispersed, interfacial, or otherwise extractable PCB fractions present under the standardized stock-preparation conditions. Application to other PCB mixtures, pure congeners, other hydrophobic organic contaminants, or substantially different concentration ranges should be separately validated. Internal-standard correction was not included in the present workflow; therefore, consistent calibration, blank control, retention-time verification, and unchanged GC-ECD operating parameters are essential. Open-vessel heating under exhaust airflow is not recommended. Stock solutions should be heated and mixed in sealed borosilicate bottles and opened only briefly during sampling. Future applications requiring full regulatory-style analytical validation may incorporate a non-target internal or surrogate standard. Applications requiring regulatory-style absolute quantification should ensure that all sample responses are bracketed by the calibration range or that extracts are diluted and reanalyzed before concentration calculation. Headspace losses were not quantified independently; therefore, users should keep bottle volume, fill volume, closure type, temperature, and agitation conditions consistent, and should separately validate the protocol if vessel geometry or headspace volume is changed. A further limitation of the present protocol is that it evaluates preparation reproducibility and solvent-extractable PCB recovery, but it does not independently quantify the freely dissolved concentration of each congener.

**Table 7:** Troubleshooting guide for stabilization of aqueous PCB stock solutions.

Observed issue	Likely cause	Recommended solution
Low or decreasing GC-ECD peak area for PCB congeners	Partitioning of PCBs to container walls during storage; incomplete short-term equilibration	Apply heating (32–34) °C combined with intensive stirring (800–900) rpm for 15–30 min prior to use; Use temperatures up to 38 °C only as an explicitly reported troubleshooting condition. Verify chromatographic response before experiments
High variability between consecutive GC injections	Inhomogeneous stock solution due to insufficient mixing	Increase stirring intensity and duration during stabilization; ensure consistent sampling location and volume
Incomplete recovery after mixing at room temperature	Insufficient thermal input to promote re-equilibration of hydrophobic PCBs	Apply mild heating during mixing (≥32 °C); avoid relying on mixing alone
Pronounced loss of PCB signal after storage in plastic containers	Sorption of PCBs to polymeric container surfaces	Prepare and store stocks in borosilicate glass bottles; avoid plastic containers for PCB stocks
Drift in apparent PCB concentration between experimental days	Re-partitioning of PCBs to container walls during storage	Reapply stabilization protocol prior to each experimental day; perform chromatographic verification before use
Poor reproducibility after heating	Non-uniform temperature control or insufficient mixing	Ensure temperature is maintained within (32–34) °C throughout stabilization; use magnetic stirring at controlled rpm
Unexpected chromatographic variability	Contamination or adsorption on glassware	Pre-rinse all glassware with distilled water followed by ethanol prior to use

## CRediT authorship contribution statement

**Renáta Števuľová:** Investigation, Methodology, Formal analysis, Data curation, Writing – review & editing. **Saimohana Krishna Vadlamudi:** Conceptualization, Methodology, Formal analysis, Writing – original draft. **Ladislav Štibrányi:** Validation, Methodology, Investigation. **Ján Híveš:** Supervision, Funding acquisition, Project administration.

## Declaration of competing interest

The authors declare that they have no known competing financial interests or personal relationships that could have appeared to influence the work reported in this paper.

## Data Availability

Data will be made available on request.

## Appendix Supplementary materials

**Supplementary material:** Supplementary tables are provided to support reproducibility and transparency of the protocol. They include raw congener-wise GC-ECD peak areas, congener-specific apparent aqueous concentrations, calibration equations, sequential extraction recovery calculations, and a supporting lower-concentration validation table. Representative chromatograms and graphical abstract files should be submitted separately if required by the journal. Image, application 1

## References

[1] Erickson M.D., Kaley R.G. (2011). Applications of polychlorinated biphenyls. Environ. Sci. Pollut. Res..

[2] Zhou Y., Miao D., Gomez-Eyles J.L., Ghosh U., Bi M., Li J., Ren F. (2022). Comparative study on polychlorinated biphenyl sorption to activated carbon and biochar and the influence of natural organic matter. Chemosphere.

[3] Wang F., Ren X., Sun H., Ma L., Zhu H., Xu J. (2016). Sorption of polychlorinated biphenyls onto biochars derived from corn straw and the effect of propranolol. Bioresour. Technol..

[4] Li N., Wania F., Lei Y.D., Daly G.L. (2003). A comprehensive and critical compilation, evaluation, and selection of physical-chemical property data for selected polychlorinated biphenyls. J. Phys. Chem. Ref. Data.

[5] Hawker D.W., Connell D.W. (1988). Octanol–water partition coefficients of polychlorinated biphenyl congeners. Environ. Sci. Technol..

[6] Wang Z., Yang K., Lin D. (2023). Adsorption and desorption of polychlorinated biphenyls on biochar colloids with different pyrolysis temperatures: the effect of solution chemistry. Environ. Sci. Pollut. Res..

[7] U.S. Environmental Protection Agency, Method 3510C: Separatory Funnel Liquid–Liquid Extraction, Test Methods for Evaluating Solid Waste: Physical/Chemical Methods, SW-846, Revision 3, Office of Solid Waste, Washington, DC, USA, December 1996. https://www.epa.gov/sites/default/files/2015-12/documents/3510c.pdf.

[8] U.S. Environmental Protection Agency, Method 8082A: Polychlorinated Biphenyls (PCBs) by Gas Chromatography, Test Methods for Evaluating Solid Waste: Physical/Chemical Methods, SW-846, Revision 1, Office of Solid Waste, Washington, DC, USA, February 2007. https://www.epa.gov/sites/default/files/2015-12/documents/8082a.pdf⁠.

[9] U.S. Environmental Protection Agency, Method 608.3: Organochlorine Pesticides and PCBs by GC/HSD, EPA-821-R-16-009, Office of Water, Washington, DC, USA, December 2016. https://www.epa.gov/cwa-methods/approved-cwa-test-methods-organic-compounds⁠.

[10] Huang Q., Hong C.-S. (2002). Aqueous solubilities of non-ortho and mono-ortho PCBs at four temperatures. Water Res..

[11] Shiu W.Y., Mackay D. (1986). A critical review of aqueous solubilities, vapor pressures, Henry’s law constants, and octanol-water partition coefficients of the polychlorinated biphenyls. J. Phys. Chem. Ref. Data.

[12] Taniyasu S., Kannan K., Holoubek I., Ansorgova A., Horii Y., Hanari N., Yamashita N., Aldous K.M. (2003). Isomer-specific analysis of chlorinated biphenyls, naphthalenes and dibenzofurans in Delor: polychlorinated biphenyl preparations from the former Czechoslovakia. Environ. Pollut..

[13] Grabic R., Hansen L.G., Ptak A., Crhova S., Gregoraszczuk E. (2006). Differential accumulation of low-chlorinated (Delor 103) and high-chlorinated (Delor 106) biphenyls in human placental tissue and opposite effects on conversion of DHEA to E2. Chemosphere.

