# Supplementary material for: Temperature-assisted stabilization of aqueous polychlorinated biphenyl stock solutions for sorption experiments
Source: MethodsX. 2026 Jun 9;17:103997. doi: 10.1016/j.mex.2026.103997 (PMC13316641; doi:10.1016/j.mex.2026.103997)
Supplement: Supplementary file 1 — Supplementary material: Supplementary tables are provided to support reproducibility and transparency of the protocol. They include raw congener-wise GC-ECD peak areas, congener-specific apparent aqueous concentrations, calibration equations, sequential extraction recovery calculations, and a supporting lower-concentration validation table. Representative chromatograms and graphical abstract files should be submitted separately if required by the journal. [file mmc1.docx]

**Temperature-assisted stabilization of aqueous polychlorinated biphenyl stock solutions for sorption experiments**

Renáta Števuľová^1*^, Saimohana Krishna Vadlamudi^1^, Ladislav Štibrányi^1^ and Ján Híveš^1^

^1^Slovak University of Technology in Bratislava, Institute of Inorganic Chemistry, Technology and Materials, Radlinského 9, 812 37 Bratislava, Slovakia

**^*^Corresponding Author:**

Renáta Števuľová

Institute of Inorganic Chemistry, Technology and Materials

Slovak University of Technology in Bratislava

Radlinského 9

812 37, Bratislava

Slovakia

Email: renata.stevulova@stuba.sk

This supplementary material provides representative chromatograms and raw GC-ECD peak area data used to evaluate the reproducibility and stability of aqueous PCB stock solutions under different handling and storage conditions
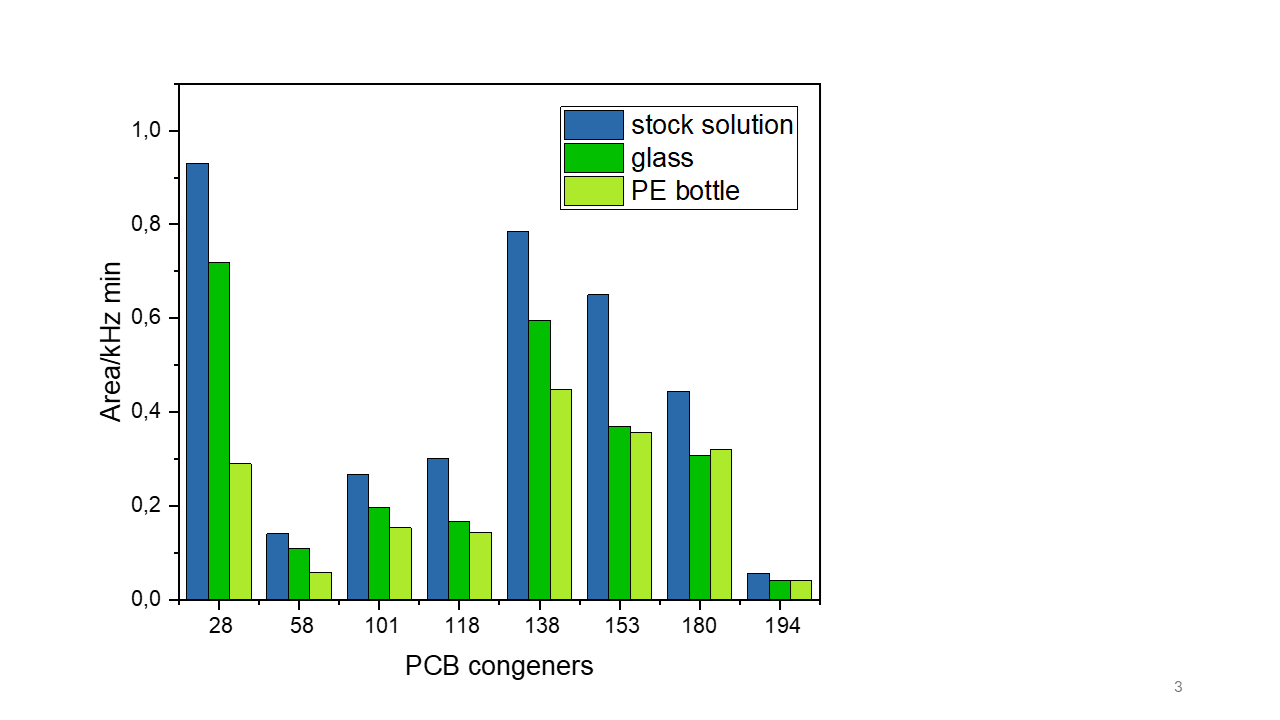


**Figure S1** Congener-specific GC-ECD peak areas of indicator PCBs measured in aqueous PCB stock solutions after 24 h storage in borosilicate glass and plastic containers. The results illustrate the influence of container material on apparent PCB concentrations.

**
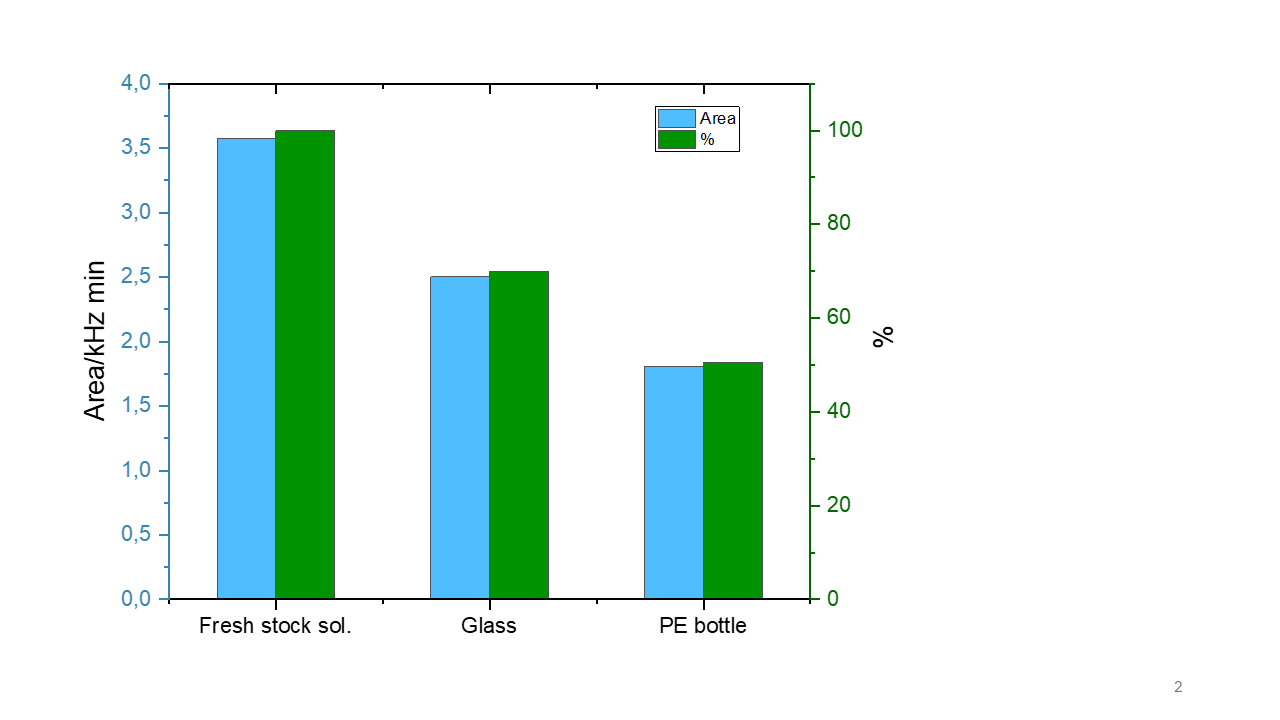
**

**Figure S2** The influence of container material on apparent PCB concentration after 24h storage, comparing borosilicate glass and plastic containers. Peak areas are expressed as the sum of the indicated PCB congeners' areas.

**Table S1. External calibration equations for the eight indicator PCB congeners.**

| **Congener** | **Intercept** | **Slope** | **R2** | **Calibration equation** |
| --- | --- | --- | --- | --- |
| PCB 28 | 0.00343 | 0.34509 | 0.99609 | Area = 0.00343 + 0.34509 x C_extract |
| PCB 52 | 0.00470 | 0.23364 | 0.98584 | Area = 0.00470 + 0.23364 x C_extract |
| PCB 101 | 0.00699 | 0.32684 | 0.98630 | Area = 0.00699 + 0.32684 x C_extract |
| PCB 118 | 0.01116 | 0.43923 | 0.97883 | Area = 0.01116 + 0.43923 x C_extract |
| PCB 138 | 0.01172 | 0.41108 | 0.97967 | Area = 0.01172 + 0.41108 x C_extract |
| PCB 153 | 0.01345 | 0.46210 | 0.97848 | Area = 0.01345 + 0.46210 x C_extract |
| PCB 180 | 0.01441 | 0.61644 | 0.98238 | Area = 0.01441 + 0.61644 x C_extract |
| PCB 194 | 0.01601 | 0.72006 | 0.97846 | Area = 0.01601 + 0.72006 x C_extract |

**Table S2. Raw GC-ECD peak areas of indicator PCB congeners used for stock validation.**

| **Condition** | **Stock** | **Replicate** | **PCB28** | **PCB52** | **PCB101** | **PCB118** | **PCB138** | **PCB153** | **PCB180** | **PCB194** | **Sigma area** |
| --- | --- | --- | --- | --- | --- | --- | --- | --- | --- | --- | --- |
| Initial stock | A |  | 1.021 | 0.144 | 0.204 | 0.157 | 0.473 | 0.357 | 0.275 | 0.034 | 2.665 |
| Initial stock | B |  | 0.956 | 0.137 | 0.201 | 0.165 | 0.489 | 0.373 | 0.297 | 0.037 | 2.655 |
| Initial stock | C |  | 1.088 | 0.156 | 0.226 | 0.184 | 0.540 | 0.060 | 0.327 | 0.040 | 2.621 |
| After storage / before stabilisation | A |  | 0.670 | 0.104 | 0.172 | 0.134 | 0.429 | 0.322 | 0.270 | 0.037 | 2.138 |
| After storage / before stabilisation | B |  | 0.542 | 0.073 | 0.132 | 0.111 | 0.384 | 0.293 | 0.261 | 0.034 | 1.830 |
| After storage / before stabilisation | C |  | 0.588 | 0.093 | 0.159 | 0.127 | 0.405 | 0.307 | 0.261 | 0.036 | 1.976 |
| Heating only | A |  | 0.687 | 0.108 | 0.185 | 0.149 | 0.473 | 0.358 | 0.302 | 0.041 | 2.303 |
| Heating only | B |  | 0.665 | 0.100 | 0.177 | 0.145 | 0.471 | 0.357 | 0.301 | 0.039 | 2.255 |
| Heating only | C |  | 0.770 | 0.126 | 0.225 | 0.185 | 0.581 | 0.067 | 0.376 | 0.051 | 2.381 |
| Heating + mixing | A |  | 0.835 | 0.137 | 0.251 | 0.213 | 0.659 | 0.508 | 0.423 | 0.053 | 3.079 |
| Heating + mixing | B |  | 0.788 | 0.120 | 0.220 | 0.181 | 0.625 | 0.458 | 0.393 | 0.055 | 2.840 |
| Heating + mixing | C |  | 0.900 | 0.143 | 0.252 | 0.205 | 0.636 | 0.424 | 0.407 | 0.055 | 3.022 |
| Before restabilisation | A | rep1 | 0.549 | 0.084 | 0.148 | 0.123 | 0.393 | 0.302 | 0.259 | 0.035 | 1.893 |
| Before restabilisation | A | rep2 | 0.499 | 0.078 | 0.137 | 0.024 | 0.303 | 0.282 | 0.242 | 0.034 | 1.599 |
| Before restabilisation | A | rep3 | 0.555 | 0.087 | 0.151 | 0.125 | 0.396 | 0.308 | 0.261 | 0.037 | 1.920 |
| Before restabilisation | B |  | 0.504 | 0.076 | 0.133 | 0.110 | 0.353 | 0.265 | 0.231 | 0.032 | 1.704 |
| Before restabilisation | C |  | 0.537 | 0.082 | 0.144 | 0.118 | 0.377 | 0.285 | 0.247 | 0.034 | 1.824 |
| After restabilisation | A | rep1 | 0.859 | 0.142 | 0.274 | 0.230 | 0.748 | 0.079 | 0.501 | 0.065 | 2.898 |
| After restabilisation | A | rep2 | 0.789 | 0.129 | 0.237 | 0.201 | 0.633 | 0.439 | 0.428 | 0.059 | 2.915 |
| After restabilisation | A | rep3 | 0.864 | 0.143 | 0.269 | 0.228 | 0.715 | 0.545 | 0.468 | 0.061 | 3.293 |
| After restabilisation | B |  | 0.799 | 0.132 | 0.250 | 0.211 | 0.663 | 0.075 | 0.426 | 0.053 | 2.609 |
| After restabilisation | C |  | 0.790 | 0.131 | 0.242 | 0.204 | 0.637 | 0.490 | 0.414 | 0.055 | 2.963 |

**Table S3. Apparent aqueous concentrations calculated from congener-specific calibration curves.**

| **Condition** | **Stock** | **Replicate** | **PCB28 water (mg/L)** | **PCB52 water (mg/L)** | **PCB101 water (mg/L)** | **PCB118 water (mg/L)** | **PCB138 water (mg/L)** | **PCB153 water (mg/L)** | **PCB180 water (mg/L)** | **PCB194 water (mg/L)** | **Sigma8PCB water (mg/L)** |
| --- | --- | --- | --- | --- | --- | --- | --- | --- | --- | --- | --- |
| Initial stock | A |  | 0.590 | 0.119 | 0.121 | 0.066 | 0.224 | 0.149 | 0.085 | 0.005 | 1.359 |
| Initial stock | B |  | 0.552 | 0.113 | 0.119 | 0.070 | 0.232 | 0.156 | 0.092 | 0.006 | 1.339 |
| Initial stock | C |  | 0.629 | 0.130 | 0.134 | 0.079 | 0.257 | 0.020 | 0.101 | 0.007 | 1.356 |
| After storage / before stabilisation | A |  | 0.386 | 0.085 | 0.101 | 0.056 | 0.203 | 0.134 | 0.083 | 0.006 | 1.054 |
| After storage / before stabilisation | B |  | 0.312 | 0.058 | 0.076 | 0.045 | 0.181 | 0.121 | 0.080 | 0.005 | 0.880 |
| After storage / before stabilisation | C |  | 0.339 | 0.076 | 0.093 | 0.053 | 0.191 | 0.127 | 0.080 | 0.006 | 0.964 |
| Heating only | A |  | 0.396 | 0.088 | 0.109 | 0.063 | 0.224 | 0.149 | 0.093 | 0.007 | 1.130 |
| Heating only | B |  | 0.383 | 0.082 | 0.104 | 0.061 | 0.223 | 0.149 | 0.093 | 0.006 | 1.101 |
| Heating only | C |  | 0.444 | 0.104 | 0.133 | 0.079 | 0.277 | 0.023 | 0.117 | 0.010 | 1.188 |
| Heating + mixing | A |  | 0.482 | 0.113 | 0.149 | 0.092 | 0.315 | 0.214 | 0.133 | 0.010 | 1.508 |
| Heating + mixing | B |  | 0.455 | 0.099 | 0.130 | 0.077 | 0.298 | 0.192 | 0.123 | 0.011 | 1.386 |
| Heating + mixing | C |  | 0.520 | 0.118 | 0.150 | 0.088 | 0.304 | 0.178 | 0.127 | 0.011 | 1.496 |
| Before restabilisation | A | rep1 | 0.316 | 0.068 | 0.086 | 0.051 | 0.186 | 0.125 | 0.079 | 0.005 | 0.916 |
| Before restabilisation | A | rep2 | 0.287 | 0.063 | 0.080 | 0.006 | 0.142 | 0.116 | 0.074 | 0.005 | 0.772 |
| Before restabilisation | A | rep3 | 0.320 | 0.070 | 0.088 | 0.052 | 0.187 | 0.127 | 0.080 | 0.006 | 0.930 |
| Before restabilisation | B |  | 0.290 | 0.061 | 0.077 | 0.045 | 0.166 | 0.109 | 0.070 | 0.004 | 0.823 |
| Before restabilisation | C |  | 0.309 | 0.066 | 0.084 | 0.049 | 0.178 | 0.118 | 0.075 | 0.005 | 0.884 |
| After restabilisation | A | rep1 | 0.496 | 0.118 | 0.163 | 0.100 | 0.358 | 0.028 | 0.158 | 0.014 | 1.434 |
| After restabilisation | A | rep2 | 0.455 | 0.106 | 0.141 | 0.086 | 0.302 | 0.184 | 0.134 | 0.012 | 1.421 |
| After restabilisation | A | rep3 | 0.499 | 0.118 | 0.160 | 0.099 | 0.342 | 0.230 | 0.147 | 0.012 | 1.608 |
| After restabilisation | B |  | 0.461 | 0.109 | 0.149 | 0.091 | 0.317 | 0.027 | 0.134 | 0.010 | 1.297 |
| After restabilisation | C |  | 0.456 | 0.108 | 0.144 | 0.088 | 0.304 | 0.206 | 0.130 | 0.011 | 1.447 |

**Table S4. Summary statistics for apparent aqueous Sigma8PCB concentrations.**

| **Condition** | **Stock A** | **Stock B** | **Stock C** | **Mean** | **SD** | **RSD (%)** |
| --- | --- | --- | --- | --- | --- | --- |
| Initial stock | 1.359 | 1.339 | 1.356 | 1.351 | 0.010 | 0.8 |
| After storage / before stabilisation | 1.054 | 0.880 | 0.964 | 0.966 | 0.087 | 9.0 |
| Heating only | 1.130 | 1.101 | 1.188 | 1.140 | 0.044 | 3.9 |
| Heating + mixing | 1.508 | 1.386 | 1.496 | 1.463 | 0.068 | 4.6 |
| Before restabilisation | 0.873 | 0.823 | 0.884 | 0.860 | 0.032 | 3.8 |
| After restabilisation | 1.488 | 1.297 | 1.447 | 1.411 | 0.100 | 7.1 |

**Table S5. Raw congener-wise areas for sequential extraction recovery testing.**

| **Condition** | **Extraction** | **PCB28** | **PCB52** | **PCB101** | **PCB118** | **PCB138** | **PCB153** | **PCB180** | **PCB194** | **Sigma area** |
| --- | --- | --- | --- | --- | --- | --- | --- | --- | --- | --- |
| Before restabilisation - Stock A | E1 | 0.609 | 0.097 | 0.171 | 0.143 | 0.455 | 0.351 | 0.300 | 0.041 | 2.167 |
| Before restabilisation - Stock A | E2 | 0.103 | 0.015 | 0.028 | 0.023 | 0.080 | 0.060 | 0.051 | 0.000 | 0.360 |
| Before restabilisation - Stock A | E3 | 0.011 | 0.000 | 0.000 | 0.000 | 0.014 | 0.000 | 0.013 | 0.000 | 0.038 |
| After restabilisation - Stock A | E1 | 0.790 | 0.131 | 0.242 | 0.204 | 0.637 | 0.490 | 0.414 | 0.055 | 2.963 |
| After restabilisation - Stock A | E2 | 0.271 | 0.044 | 0.086 | 0.071 | 0.231 | 0.175 | 0.145 | 0.019 | 1.042 |
| After restabilisation - Stock A | E3 | 0.069 | 0.010 | 0.022 | 0.019 | 0.065 | 0.049 | 0.041 | 0.000 | 0.275 |

**Table S6. Congener-specific concentrations in cyclohexane extracts during recovery testing.**

| **Condition** | **Extraction** | **PCB28 extract (mg/L)** | **PCB52 extract (mg/L)** | **PCB101 extract (mg/L)** | **PCB118 extract (mg/L)** | **PCB138 extract (mg/L)** | **PCB153 extract (mg/L)** | **PCB180 extract (mg/L)** | **PCB194 extract (mg/L)** | **Sigma8PCB extract (mg/L)** |
| --- | --- | --- | --- | --- | --- | --- | --- | --- | --- | --- |
| Before restabilisation - Stock A | E1 | 1.755 | 0.395 | 0.502 | 0.300 | 1.078 | 0.730 | 0.463 | 0.035 | 5.259 |
| Before restabilisation - Stock A | E2 | 0.289 | 0.044 | 0.064 | 0.027 | 0.166 | 0.101 | 0.059 | 0.000 | 0.750 |
| Before restabilisation - Stock A | E3 | 0.022 | 0.000 | 0.000 | 0.000 | 0.006 | 0.000 | 0.000 | 0.000 | 0.027 |
| After restabilisation - Stock A | E1 | 2.279 | 0.541 | 0.719 | 0.439 | 1.521 | 1.031 | 0.648 | 0.054 | 7.233 |
| After restabilisation - Stock A | E2 | 0.775 | 0.168 | 0.242 | 0.136 | 0.533 | 0.350 | 0.212 | 0.004 | 2.421 |
| After restabilisation - Stock A | E3 | 0.190 | 0.023 | 0.046 | 0.018 | 0.130 | 0.077 | 0.043 | 0.000 | 0.526 |

**Table S7. Sequential extraction recovery summary.**

| **Condition** | **E1 Sigma8PCB extract (mg/L)** | **E2 Sigma8PCB extract (mg/L)** | **E3 Sigma8PCB extract (mg/L)** | **Apparent aqueous total (mg/L)** | **E1 recovery (%)** | **E1+E2 recovery (%)** |
| --- | --- | --- | --- | --- | --- | --- |
| Before restabilisation - Stock A | 5.259 | 0.750 | 0.027 | 1.207 | 87.1 | 99.5 |
| After restabilisation - Stock A | 7.233 | 2.421 | 0.526 | 2.036 | 71.1 | 94.8 |

**Table S8. Supporting lower-concentration check at nominal 0.5 mg L^-1^ PCB stock concentration.**

| **Condition** | **Measurement** | **Ʃ8PCB response (area)** | **Converted concentration** | **Note** |
| --- | --- | --- | --- | --- |
| 0.5 mg/L stock before stabilization | Single/representative | 1.0 | - | Supporting total-area observation |
| 0.5 mg/L stock after stabilization | Replicate 1 | 3.2 | - | Supporting total-area observation |
| 0.5 mg/L stock after stabilization | Replicate 2 | 3.4 | - | Supporting total-area observation |
| 0.5 mg/L stock after stabilization | Replicate 3 | 3.6 | - | Supporting total-area observation |
| 0.5 mg/L stock after stabilization | Mean +/- SD | 3.4 +/- 0.2 | - | Area-based supporting check only |

**Table S9. Sensitivity check excluding PCB153 (Ʃ7PCB apparent aqueous concentration).**

| **Condition** | **Stock A** | **Stock B** | **Stock C** | **Mean** | **SD** | **RSD (%)** |
| --- | --- | --- | --- | --- | --- | --- |
| Initial stock | 1.210 | 1.184 | 1.336 | 1.243 | 0.081 | 6.5 |
| After storage / before stabilisation | 0.920 | 0.759 | 0.837 | 0.839 | 0.081 | 9.6 |
| Heating only | 0.981 | 0.953 | 1.165 | 1.033 | 0.115 | 11.1 |
| Heating + mixing | 1.294 | 1.193 | 1.318 | 1.268 | 0.066 | 5.2 |
| Before restabilisation | 0.750 | 0.714 | 0.766 | 0.743 | 0.027 | 3.6 |
| After restabilisation | 1.340 | 1.270 | 1.240 | 1.284 | 0.051 | 4.0 |

Notes: Values below calibration intercept were treated as zero. Apparent aqueous concentrations were calculated from the extraction ratio of 50 mL aqueous sample to 10 mL cyclohexane extract. For restabilisation rows, Stock A values represent means of triplicate measurements before comparison with Stocks B and C. The 0.5 mg L-1 lower-concentration check is included as supporting total-area evidence only because congener-wise calibration data were not available for that check.
